# The Performance of Primary Healthcare in China: The Need for a Systematic Design for Improvement: Comment on "Quality and Performance Measurement in Primary Diabetes Care: A Qualitative Study in Urban China"

**DOI:** 10.34172/ijhpm.2023.7889

**Published:** 2023-05-10

**Authors:** Weiyan Jian, Qiaosheng Li, Lanyue Zhang

**Affiliations:** Department of Health Policy and Management, School of Public Health, Peking University, Beijing, China

**Keywords:** Primary Healthcare, Quality of Care, China

## Abstract

In the paper "Quality and Performance Measurement in Primary Diabetes Care: A Qualitative Study in Urban China," Rasooly and colleagues provide an in-depth analysis of the ways in which Shanghai manages the quality and performance of the primary healthcare (PHC). The present commentary extends the analytical perspective offered in this paper from the city of Shanghai to the entire Chinese Mainland. In so doing, it points out certain systemic shortcomings in the capabilities of family doctors, the unreasonable competition between primary, secondary, and tertiary forms of healthcare, and the negative incentives in the salary system for PHC providers that must be overcome to improve performance. This commentary also proposes strategies and other recommendations for overcoming the bottlenecks identified in the paper as a means of systematically enhancing PHC performance across Mainland China.

## Introduction

 In their paper, Rasooly and colleagues examine 26 key primary healthcare (PHC) stakeholders’ perceptions of quality and performance measurements in Shanghai, China, selecting primary diabetes care as their central case in this consideration of the factors facilitating and impeding implementation.^1^ A well-established PHC system is essential for achieving universal healthcare coverage, especially in the case of a country with a huge population and relatively inadequate healthcare resources.

 Over the past 20 years, the Chinese government has continued to increase funding for PHC institutions, from 19 billion RMB in 2008 to 197 billion RMB in 2018.^2^ The infrastructure associated with PHC institutions has also been strengthened considerably.^3^ At the same time, the number of PHC providers in China has continuously grown, with the total number of general practitioners (GPs) increasing from 109 800 to 434 900 in the last decade, and the number of GPs per 10 000 population increasing from 0.81 to 3.08. Based on such continuous increase in total PHC resources, improvements have been witnessed in terms of the resource-based equity of PHC between regions, with differences between the eastern and central and western regions gradually narrowing. By 2021, the number of GPs per 10 000 population had increased to 3.69 in the eastern region, and to 2.71 and 2.53 in the central and western regions respectively, up from the 2012 figures of 1.19, 0.52, and 0.58,^4^ respectively, at which point the difference between the eastern and central and western regions was more than double.

 Despite such progress, to date, the overall performance of the PHC service system in China remains unsatisfactory. Despite the Chinese government’s continued investment in PHC facilities over the past 10 years, patients are still concentrated in tertiary hospitals. Taking outpatient services as an example, the proportion of outpatient appointments rose from 39.9% in 2009 to 60.3% in 2021.^4^ At the same time, high and avoidable hospitalization rates of Chinese residents, including the patients with diabetes that Rasooly and colleagues are primarily concerned with, have shown little sign of declining in recent years,^5,6^ providing further evidence of poor PHC provision.

## Difficulties and Challenges With Developing PHC in China

 With the improvement of PHC facilities and human resources, the key to making improvements in the performance of PHC in China is to enhance the capacity and motivation of family doctors.^7^ The “PHC quality and performance measurement” which focused in Rasooly and colleagues’ article is an important incentive affecting the capacity and motivation of such family doctors. Moreover, when one takes a high-level view of the Mainland Chinese PHC system, in addition to the problems identified by Rasooly and colleagues (eg, frontline clinicians from indicator planning, a lack of transparent reporting, and a rigid organizational culture with limited bottom-up feedback), there are yet further and deeper issues that must be considered.

 First, the establishment of the academic discipline studied by Chinese GPs occurred at a relatively late stage. This is especially true of economically underdeveloped areas, where most “family doctors” still do not receive systematic training in general medicine.^2,8^ Moreover, the career development paths of GPs remain unclear, resulting in the field remaining less attractive to suitable candidates than would be ideal.^9^ Extant studies also provide evidence that members of staff working in Chinese family doctor teams face serious problems in terms of professional burnout. Indeed, the proportion of the professional medical population dealing with professional burnout is reported as being as high as 73.6%.^10^ There are also reports of instability in team structure, with unclear career paths having a significant negative impact on medical students’ willingness to practice in PHC institutions. Only 29.9% of graduated medical students are noted as being willing to work in such PHC institutions.^11^ All of these issues continue to affect the professionalism and stability of China’s family doctor teams.

 Second, China’s healthcare system is severely fragmented, and competition has arisen between primary care and secondary or tertiary care, characterized by a lack of cooperative mechanisms between these different sectors.^12^ Without the institutional guarantee of family doctors as “gatekeepers,” PHC providers are at disadvantage in terms of the competition with secondary and tertiary care providers. Many patients are siphoned off to high-level hospitals, exacerbating the recent problem of the continued concentration of outpatients in Chinese tertiary hospitals noted earlier. A common problem faced by family doctors is a lack of patient volume as a basic guarantee of the capacity of healthcare providers. For instance, the daily rate of doctors treating patients in PHC institutions is only a quarter of that in tertiary hospitals.^4^ Without sufficient patient volume, it is also difficult to improve the capacity of family doctors. Extant research reports that local PHC facilities correctly diagnosed 44.11% of standardized patients with unstable angina and asthma in a developed city in China, with a correct rate of treatment of only 24.19%.^13^ This is evidence that the capacity of family doctors is less than satisfactory. Furthermore, this situation also affects demand in PHC providers, leading to a lack of confidence in the services of PHC providers. One study conducted using data from Shanghai patients finds that patients’ trust in family doctors is low, with only 25.3% of patients stating that they had a high level of trust in their family doctor, and many reporting significant concerns about the capacity and treatment-based options of family doctors.^14^ This constitutes clear evidence of a vicious cycle: “low trust – fewer patients – decreased ability – even lower trust – even fewer patients – even poorer ability.”

 The third problem to note at this point relates to the salary system for Chinese family doctors. This is unfavorable as a means of incentivizing the improvement of capacity and performance. The interview-obtained data provided by Rasooly et al suggest that Shanghai may have made efforts in this regard by designing bonuses based on performance with a view to positively influencing family doctors’ behavior. However, there remain a number of areas in China where family doctors are paid a fixed salary independent of the quantity and quality of service provided.^15^ In most areas where a performance-based salary system is implemented, performance-based salary only accounts for a small proportion of family doctors’ income, preventing positive and larger-scale manipulation of family doctors’ motivation to deliver the best healthcare. Coupled with the various problems affecting the process of quality and performance measurement mentioned by Rasooly and colleagues, the end result is little or no difference in income between family doctors who demonstrate superior performance and those who demonstrate poor performance,^16^ resulting in a lack of opportunity to positively influence such medical professionals’ conduct.

## Paths to Improve PHC Performance in China

 Given the overall situation affecting PHC in Mainland China, in addition to the recommendations proposed by Rasooly and colleagues, the following three measures should also be considered as a means of improving the performance of Chinese PHC:

###  (1) Improving the Capacity of PHC Providers Through Artificial Intelligence 

 Rasooly and colleagues note that their investigation of the Shanghai system found that specialist doctors in hospitals require the assistance of family doctors to manage patients with diabetes, resulting in the provision of training to the latter to enhance their abilities. However, it should also be noted that this kind of training is not a stable and systematic arrangement that meets the needs of family doctors in terms of increasing their capacity. On this basis, it is necessary to explore alternative paths to enhance the capabilities of family doctors, and using artificial intelligence (AI) technology to assist family doctors in diagnosing, treating and managing patients with common diseases is a feasible solution. The diagnosis and treatment of common diseases, as well as the management of chronic patients, have been practiced for a long time and have sufficient evidence, making them easy for AI to learn and absorb. As a result, such work is a strong potential candidate for AI as a means of providing assistance to family doctors. If done correctly, such implementation of AI has the potential to assist family doctors reduce misdiagnosis and medical errors and thus improve their effectiveness of managing patients. This, in turn, will improve the reputation of PHC providers among those making use of their services.

###  (2) Establishing “Health Responsibility System” by Reforming the Salary System for Family Doctors

 Fostering greater levels of responsibility among family doctors in relation to the health of their service population is a fundamental approach to be taken in the larger project of improving PHC performance. Following enhancement of their capabilities, family doctors should be positioned to gradually take on the role of gatekeepers (treating mild illness, referring appropriately severe cases of illness to specialists, managing patients with chronic conditions, etc) and their payment system should be changed such that it is based on the service population capitations. Under capitation, the healthier the service population, the higher the family doctor’s income. This will motivate family doctors to arrange healthcare from the perspective of population health, rather than in the manner referred to as “gaming the system” by Rasooly and colleagues. To ensure the smooth operation of such a system, it will be necessary to include supporting systems relating to the following goals, among others: (1) allowing community residents to fully understand and then freely choose or change their family doctors; (2) stopping the use of easily gamed indicators such as “diabetes management rate” to assess the performance of PHCs, and instead using indicators that are more relevant to residents’ health, such as encouraging PHC providers to identify patients in need of management and to reduce rates of avoidable hospitalization; (3) giving additional bonuses to family doctors if their work reduces the volume of specialty outpatient and inpatient services for community residents.

###  (3) Integrating PHC Fundraising to Foster a Joint Effort

 In China, there are two main sources of financing for supporting family doctors in providing PHC: public health funding through public finance, which compensates for the expenditure entailed by providing public health services, and basic medical insurance funds, which are used to reimburse for the expenditure of diagnosing and treating specific diseases like diabetes. In the case of primary diabetes care considered by Rasooly and colleagues, work undertaken in respect of diagnosis and treatment is remunerated by means of basic medical insurance, while the management of diabetic patients is supported by public health funding. Only partial fulfilment of the responsibilities of PHC can be achieved by the individual use of either public health funds or medical insurance funds. Integrating these two funding channels would combine the public health services and medical services into a complete service package for family doctors to provide to community residents. The resulting capitation would be close to the entire income of family doctors rather than just a part of it. In this way, it would be possible to increase the motivational impact on family doctors and thereby improve PHC performance.

## Conclusion

 To achieve the goal of “Healthy China 2030,” the key is ensuring a high level of performance in the PHC system. The path to improving PHC performance, in turn, is to leverage modern information technology to enhance PHC service capabilities on the basis of past investments in PHC infrastructure and human resources, and to create a system environment more conducive to family doctors taking greater responsibility for the health of community residents in a positive and accountable manner.

## Ethical issues

 Not applicable.

## Competing interests

 Authors declare that they have no competing interests.
